# Synthesis and crystal structure of (*E*)-1,2-bis­[2-(methyl­sulfan­yl)phen­yl]diazene

**DOI:** 10.1107/S2056989019014592

**Published:** 2019-10-31

**Authors:** Jonas Hoffmann, Thomas J. Kuczmera, Enno Lork, Anne Staubitz

**Affiliations:** a Institute for Organic and Analytic Chemistry, University Bremen, Leobener Strasse 7, 28359 Bremen, Germany; b MAPEX, Center for Materials and Processes, University of Bremen, Bibliothekstr. 1, 28359 Bremen, Germany; c Institute for Inorganic Chemistry and Crystallography, University of Bremen, Leobener Strasse 7, 28359 Bremen, Germany

**Keywords:** crystal structure, azo­benzene, *ortho*-substitution, thiols, transmetallation, Hirshfeld surface analysis

## Abstract

The title compound, C_14_H_14_N_2_S_2_, was obtained by transmetallation of 2,2′-bis­(tri­methyl­stann­yl)azo­benzene with methyl lithium, and subsequent quenching with dimethyl di­sulfide. The asymmetric unit comprises two half-mol­ecules, the other halves being completed by inversion symmetry at the midpoint of the azo group. The two mol­ecules show only slight differences with respect to N=N, S—N and aromatic C=C bonds or angles. Hirshfeld surface analysis reveals that except for one weak H⋯S inter­action, inter­molecular inter­actions are dominated by van der Waals forces only.

## Abstract 2

In the crystal structure of the title compound, two half-mol­ecules are found in the asymmetric unit. The completed mol­ecules differ only slightly in bond lengths and torsion angles.

## Chemical context   

The mol­ecular switch azo­benzene can undergo isomerization from its thermodynamically stable *trans* form to the metastable *cis* form using external stimuli such as light, temperature or pressure. Azo­benzenes are common motifs in dyes because of their high thermal and photochemical stability (Yesodha *et al.*, 2004 ▸; Lagrasta *et al.*, 1997 ▸). We recently presented methods to substitute azo­benzenes in the *ortho*, *meta* and *para*-positions with tri­methyl­tin as a novel functionalization method, giving rise to a dual tin–lithium exchange (Strüben *et al.*, 2014 ▸, 2015 ▸; Hoffmann *et al.*, 2019 ▸). In particular, we described the effect on the di*ortho*-substitution on azo­benzenes with trimethyl-tetrels and the resulting effects on the switching properties (Hoffmann *et al.*, 2019 ▸). In this context, we present here a novel di*ortho*-substituted azo­benzene, (C_7_H_7_NS)_2_, (I), bearing two methyl­sulfide groups.
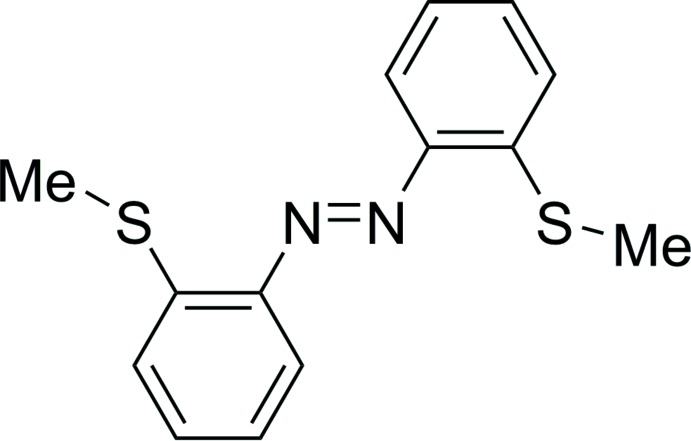


## Structural commentary   

**Figure 1:**
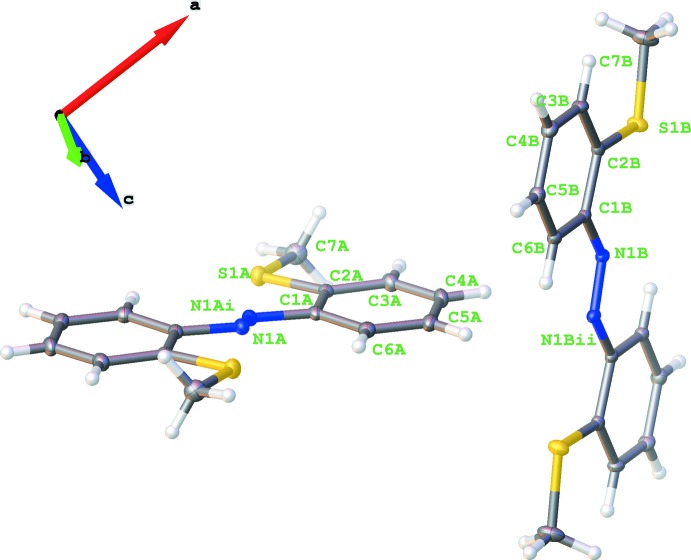
Mol­ecular structures (**Ia** left, **Ib** right) of the title compound with labelling and displacement ellipsoids drawn at the 50% probability level. [Symmetry codes: (i) *x*, 1 − *y*, − *z*; (ii) 1 − *x*, 1 − *y*, 1 − *z*.]

**Figure 2:**
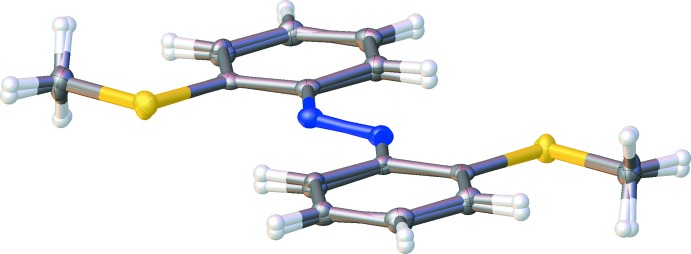
Overlay presentation of mol­ecules **Ia** and **Ib**.

The asymmetric unit of the title compound consists of two half-mol­ecules (**Ia** and **Ib**), the other halves being completed by application of inversion symmetry. The midpoints of the N=N bonds are located on inversion centres, resulting in a *trans*-configuration for the central N=N bonds (Fig. 1 ▸). As indicated by the C6*A*—C1*A*—N1*A*—N1*A* ^i^ and C6*B*—C1*B*—N1*B*—N1*B* ^ii^ [symmetry codes: (i) −*x*, 1 − *y*, −*z*; (ii) 1 − *x*, 1 − *y*, 1 − *z*] torsion angles of 13.2 (2) and −5.3 (2)°, respectively, in both mol­ecules the phenyl rings are twisted slightly with respect to the azo unit. A weak distortion is also found for the N1—C1—C2—S1 torsion angles of −3.06 (16)° for **Ia** and −2.06 (15)° for **Ib**. The N=N bond lengths differ marginally [1.255 (2) Å for **Ia**, 1.264 (2) Å for **Ib**], as do comparable C—C bonds. For example, the C1—C2 bond in **Ia** is at 1.408 (2) Å slightly shorter than **Ib** [1.415 (2) Å]. In comparison, this bond is longer than all other C—C distances in the ring because of repulsion of the nitro­gen and the sulfur atoms attached to C1 and C2, respectively. In both mol­ecules, the S⋯N distances [2.8625 (13) Å for **Ia**, 2.8761 (11) Å for **Ib**] are too long to be considered as attractive inter­actions. Fig. 2 ▸ represents an overlay plot of the two mol­ecules, showing there are only slight conformational differences.

## Supra­molecular features and Hirshfeld surface analysis   

**Figure 3:**
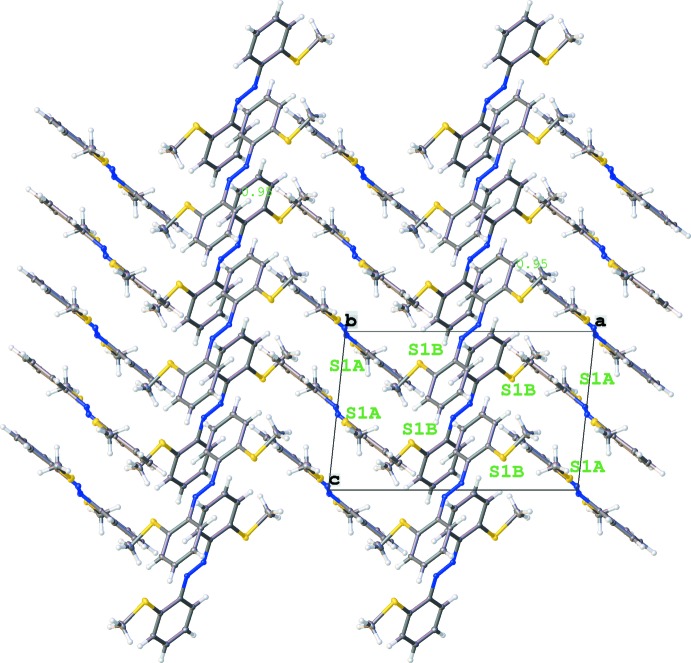
Crystal packing in a view along the *b* axis. To distinguish the different mol­ecules, all sulfur atoms within the unit cell are labelled.

**Figure 4:**
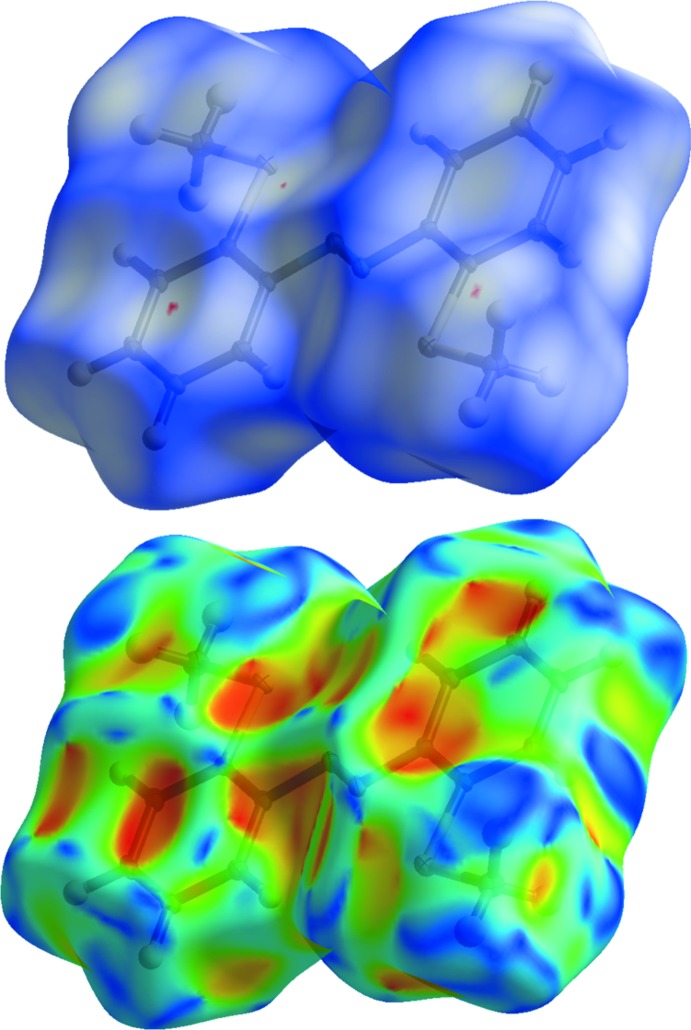
Hirshfeld surface of **Ia** mapped with *d*_norm_ (top) and shape index (bottom), displaying no significant inter­molecular inter­actions.

**Figure 5:**
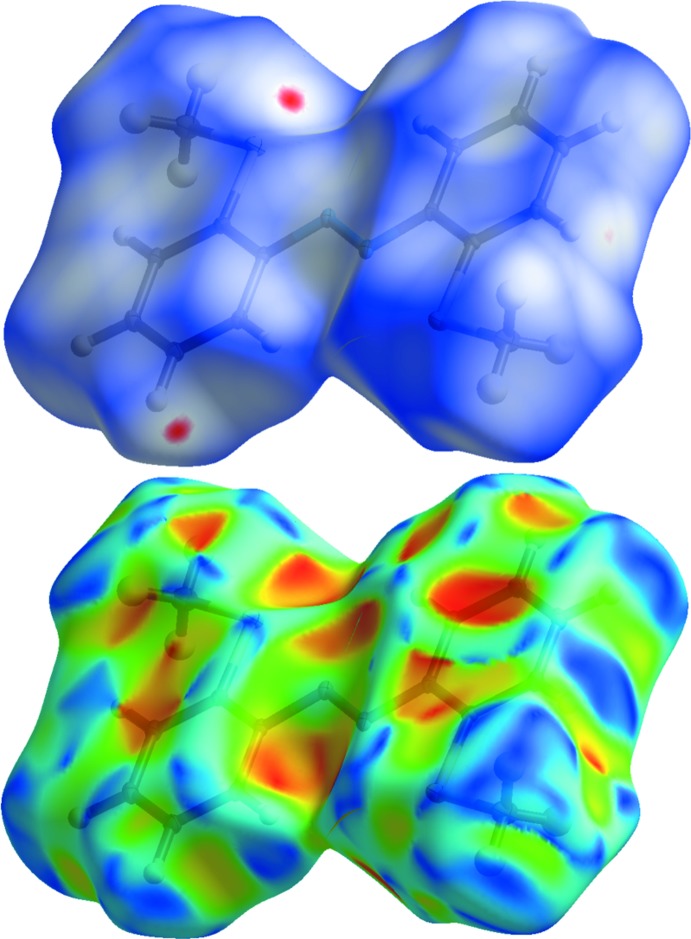
Hirshfeld surface of **Ib** mapped with *d*_norm_ (top) and shape index (bottom) with indication of an S⋯H inter­action.

**Figure 6:**
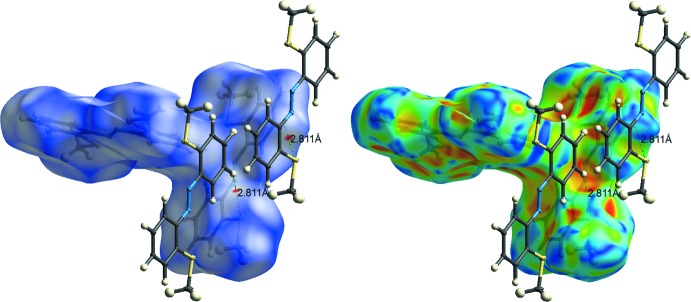
Hirshfeld surface of **Ib** mapped with *d*_norm_ (left) and shape index (right), together with the inter­action of a neighbouring mol­ecule.

**Figure 7:**
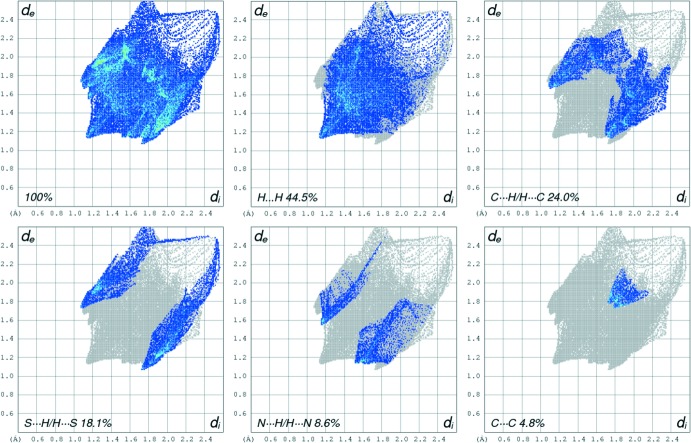
Two-dimensional fingerprint plots for **Ib**, delineated into H⋯H, C⋯H, S⋯H, N⋯H, C⋯C inter­actions.

The packing of **Ia** and **Ib** in the crystal is shown in Fig. 3 ▸. Despite the presence of phenyl rings and a parallel arrangement of the mol­ecules, only weak offset π–π inter­actions are observed; the shortest centroid-to-centroid distance is *Cg*2⋯*Cg*2(1 − *x*, 1 − *y*, −*z*) = 3.7525 (8) Å with a slippage of 1.422 Å. To further investigate the inter­molecular inter­actions, Hirshfeld surfaces (Hirshfeld, 1977 ▸) and fingerprint plots were generated for both mol­ecules using *CrystalExplorer17.5* (McKinnon *et al.*, 2004 ▸). Hirshfeld surface analysis depicts inter­molecular inter­actions by different colors, representing short or long contacts and further the relative strength of the inter­action. The generated Hirshfeld surfaces mapped over *d*_norm_ and the shape index are shown in Fig. 4 ▸ for **Ia** and in Fig. 5 ▸ for **Ib**. Whereas in **Ia** a significant inter­molecular inter­action is not apparent, characteristic red spots near S1*B* and H5*B* indicate weak S⋯H inter­actions in **Ib**. The respective supra­molecular arrangement is shown in Fig. 6 ▸. The sulfur atom S1*B* inter­acts with a phenyl proton (H4*B*) of another mol­ecule of **Ib** (S⋯H distance = 2.811 Å). The two-dimensional fingerprint plots for mol­ecule **Ib** for qu­anti­fication of the contributions of each type of non-covalent inter­action to the Hirshfeld surface (McKinnon *et al.*, 2007 ▸) are given in Fig. 7 ▸. The packing is dominated by H⋯H contacts, representing van der Waals inter­actions (44.5% contribution to the surface), followed by C⋯H and S⋯H inter­actions, which contribute with 24.0% and 18.1%, respectively. The contributions of the N⋯H (8.6%) and C⋯C (4.8%) inter­actions are less significant.

## Database survey   

A search of the Cambridge Structural Database (CSD version 5.4.0; update August 2019; Groom *et al.*, 2016 ▸) revealed no azo­benzene-based structures that contain methyl thio­ethers. However, some general *ortho*-substituted azo­benzenes have been deposited (Yamamura *et al.*, 2008 ▸; Kano *et al.*, 2001 ▸; Hoffmann *et al.*, 2019 ▸). Additionally, some di*ortho*-substituted thio­azoxybenzenes were reported previously (Szczygelska-Tao *et al.*, 1999 ▸; Kertmen *et al.*, 2013 ▸). For the structure of an azo­benzene compound with an inversion centre at the N=N bond, see: Bohle *et al.* (2007 ▸).

## Synthesis and crystallization   

The synthesis of 2,2′-*bis*(tri­methyl­stann­yl)azo­benzene was recently described (Hoffmann *et al.*, 2019 ▸). For further details of a similar transmetallation of a stannylated azo­benzene, see: Strüben *et al.* (2015 ▸). Dimethyl di­sulfide (99%) was purchased from Acros Organics and was used without further purification. Methyl lithium (1.88 *M* in diethyl ether, titrated against 2,2′-bi­pyridine) was purchased from Acros Organics. THF was purchased from VWR and was dried and degassed with a solvent purification system by Inert Technology.

**2,2′-bis­(Methyl­thio)­azo­benzene**

In an inert reaction tube, 2,2-*bis*(tri­methyl­stann­yl)azo­benzene (200 mg, 0.39 mmol) was dissolved under Schlenk conditions in THF (12.5 ml) and cooled to 195 K. Then MeLi (1.88 *M* in diethyl ether, 0.63 ml, 1.18 mmol) was added within 5 min and after 1.5 h at this temperature, dimethyl di­sulfide (0.35 ml, 3.94 mmol) was added in one ration. The reaction mixture was warmed to 298 K over 14 h and the solvent was removed under reduced pressure. The obtained orange solid was purified in a silica column (Merck, 0.015–0.40 mm) with a gradient of eluents from *n*-pentane to di­chloro­methane giving dark-orange crystals (31 mg, 0.11 mmol; yield 29%). Single crystals suitable for X-ray analysis were obtained by slow evaporation from a saturated *n*-heptane solution.

**^1^H NMR** (500 MHz, CDCl_3_): δ = 7.76 (*dd*, ^3^ *J* = 8.1 Hz, ^4^ *J* = 1.4 Hz, 2H, *H*6), 7.40 (*td*, ^3^ *J* = 8.0, 7.3 Hz, ^4^ *J* = 1.4 Hz, 2H, *H*4), 7.32 (*dd*, ^3^ *J* = 8.0 Hz, ^4^ *J* = 1.1 Hz, 2H, *H*3), 7.20 (*td*, ^3^ *J* = 8.1, 7.3Hz, ^4^ *J* = 1.1 Hz, 2H, *H*5), 2.53 (*s*, 6H, *H*7) ppm.

**^13^C{^1^H} NMR** (125 MHz, CDCl_3_): δ = 149.08 (*C*1), 141.00 (*C*2), 131.56 (*C*4), 124.81 (*C*3), 124.75 (*C*5), 118.02 (*C*6), 15.02 (*C*7) ppm.

**HRMS** (EI, 70 eV, MAT95, direct): *m*/*z*: calculated for C_14_H_14_N_2_S_2_^+^ 274.05929 found 274.05944.

**MS** (EI): *m*/*z* 273.9 (5%) [*M*]^+^, 258.9 (100%) [*M* − CH_3_]^+^, 243.9 (5%) [*M* − C_2_H_6_]^+^, 107.9 (13%) [*M* − C_8_H_10_N_2_S]^+^.

**IR** (ATR): ν = 3059 (*w*), 2986 (*w*), 2961 (*w*), 2918 (*w*), 2852 (*w*), 1575 (*m*), 1561 (*w*), 1457 (*m*), 1433 (*s*), 1298 (*w*), 1249 (*w*), 1217 (*m*), 1162 (*m*), 1065 (*s*), 1035 (*m*), 951 (*m*), 863 (*w*), 803 (*w*), 761 (*s*), 726 (*s*), 674 (*s*) cm^−1^.

**M.p.**: 429 K

***R*** ***_f_***: (*n*-penta­ne: di­chloro­methane 3:1): 0.55.

## Refinement   

Crystal data, data collection and structure refinement details are summarized in Table 1 ▸. All H atoms were positioned geometrically and refined using a riding model: C—H = 0.95–0.98 Å with *U*_iso_(H) = 1.5*U*_eq_ (C-meth­yl) and 1.2*U*_eq_(C) (C-phen­yl).

## Supplementary Material

Crystal structure: contains datablock(s) I. DOI: 10.1107/S2056989019014592/wm5522sup1.cif

Structure factors: contains datablock(s) I. DOI: 10.1107/S2056989019014592/wm5522Isup2.hkl

CCDC references: 1961741, 1961741

Additional supporting information:  crystallographic information; 3D view; checkCIF report

## Figures and Tables

**Table 1:** Experimental details

Crystal data	
Chemical formula	C_14_H_14_N_2_S_2_
*M*_r_	274.39
Crystal system, space group	Monoclinic, *P*2_1_/*c*
Temperature (K)	100
*a*, *b*, *c* (Å)	13.0656 (5), 12.1787 (4), 8.3471 (3)
β (°)	96.154 (1)
*V* (Å^3^)	1320.55 (8)
*Z*	4
Radiation type	Mo *K*α
μ (mm^−1^)	0.39
Crystal size (mm)	0.21 × 0.18 × 0.17

Data collection	
Diffractometer	Bruker D8 Venture CMOS
Absorption correction	Multi-scan (*SADABS*; Krause *et al.*, 2015 ▸)
*T*_min_, *T*_max_	0.580, 0.746
No. of measured, independent and observed [*I* > 2σ(*I*)] reflections	21000, 3292, 2842
*R*_int_	0.065
(sin θ/λ)_max_ (Å^−1^)	0.668

Refinement	
*R*[*F* ^2^ > 2σ(*F* ^2^)], *wR*(*F* ^2^), *S*	0.034, 0.089, 1.04
No. of reflections	3292
No. of parameters	165
H-atom treatment	H-atom parameters constrained
Δρ_max_, Δρ_min_ (e Å^−3^)	0.44, −0.38

Computer programs: *APEX3* and *SAINT* (Bruker, 2016 ▸), *SHELXT* (Sheldrick, 2015*a* ▸), *SHELXL* (Sheldrick, 2015*b* ▸) and *OLEX2* (Dolomanov *et al.*, 2009 ▸).

## supplementary crystallographic information

### Crystal data

**Table:**

C_14_H_14_N_2_S_2_	*F*(000) = 576
*M**_r_* = 274.39	*D*_x_ = 1.380 Mg m^−^^3^
Monoclinic, *P*2_1_/*c*	Mo *K*α radiation, λ = 0.71073 Å
*a* = 13.0656 (5) Å	Cell parameters from 9874 reflections
*b* = 12.1787 (4) Å	θ = 2.3–28.3°
*c* = 8.3471 (3) Å	µ = 0.39 mm^−^^1^
β = 96.154 (1)°	*T* = 100 K
*V* = 1320.55 (8) Å^3^	Block, dark orange
*Z* = 4	0.21 × 0.18 × 0.17 mm

### Data collection

**Table:**

Bruker D8 Venture CMOS diffractometer	3292 independent reflections
Radiation source: microfocus sealed X-ray tube, Incoatec Iµs	2842 reflections with *I* > 2σ(*I*)
Mirror optics monochromator	*R*_int_ = 0.065
Detector resolution: 7.9 pixels mm^-1^	θ_max_ = 28.3°, θ_min_ = 2.3°
ω and φ scans	*h* = −17→17
Absorption correction: multi-scan (*SADABS*; Krause *et al.*, 2015)	*k* = −16→16
*T*_min_ = 0.580, *T*_max_ = 0.746	*l* = −11→10
21000 measured reflections	

### Refinement

**Table:**

Refinement on *F*^2^	Primary atom site location: dual
Least-squares matrix: full	Hydrogen site location: inferred from neighbouring sites
*R*[*F*^2^ > 2σ(*F*^2^)] = 0.034	H-atom parameters constrained
*wR*(*F*^2^) = 0.089	*w* = 1/[σ^2^(*F*_o_^2^) + (0.0401*P*)^2^ + 0.6716*P*] where *P* = (*F*_o_^2^ + 2*F*_c_^2^)/3
*S* = 1.03	(Δ/σ)_max_ < 0.001
3292 reflections	Δρ_max_ = 0.44 e Å^−^^3^
165 parameters	Δρ_min_ = −0.38 e Å^−^^3^
0 restraints	

### Special details

**Table:**

Geometry. All esds (except the esd in the dihedral angle between two l.s. planes) are estimated using the full covariance matrix. The cell esds are taken into account individually in the estimation of esds in distances, angles and torsion angles; correlations between esds in cell parameters are only used when they are defined by crystal symmetry. An approximate (isotropic) treatment of cell esds is used for estimating esds involving l.s. planes.

### Fractional atomic coordinates and isotropic or equivalent isotropic displacement parameters (Å^2^)

**Table:**

	*x*	*y*	*z*	*U*_iso_*/*U*_eq_	
S1A	0.03738 (3)	0.22038 (3)	0.07109 (4)	0.01750 (10)	
N1A	0.00557 (9)	0.45111 (10)	0.02308 (14)	0.0144 (2)	
C1A	0.09596 (10)	0.43239 (11)	0.13020 (16)	0.0133 (3)	
C2A	0.12139 (10)	0.32133 (11)	0.15962 (16)	0.0136 (3)	
C3A	0.21227 (10)	0.29785 (12)	0.25937 (17)	0.0163 (3)	
H3A	0.232873	0.223733	0.278065	0.020*	
C4A	0.27191 (10)	0.38209 (12)	0.33050 (17)	0.0175 (3)	
H4A	0.333363	0.364884	0.397179	0.021*	
C5A	0.24372 (10)	0.49160 (12)	0.30637 (17)	0.0170 (3)	
H5A	0.284385	0.548538	0.358432	0.020*	
C6A	0.15556 (10)	0.51642 (12)	0.20540 (16)	0.0153 (3)	
H6A	0.135717	0.590824	0.187411	0.018*	
C7A	0.09736 (12)	0.09607 (12)	0.15119 (19)	0.0212 (3)	
H7AA	0.108296	0.100874	0.268985	0.032*	
H7AB	0.163749	0.086347	0.108405	0.032*	
H7AC	0.052690	0.033332	0.119805	0.032*	
S1B	0.68646 (3)	0.36826 (3)	0.29648 (4)	0.01522 (10)	
N1B	0.53057 (8)	0.47643 (9)	0.45728 (13)	0.0111 (2)	
C1B	0.54712 (9)	0.53356 (10)	0.31394 (15)	0.0107 (2)	
C2B	0.62096 (9)	0.48706 (11)	0.22188 (15)	0.0109 (2)	
C3B	0.63884 (10)	0.53770 (11)	0.07713 (16)	0.0135 (3)	
H3B	0.688084	0.507481	0.013715	0.016*	
C4B	0.58521 (10)	0.63170 (11)	0.02550 (16)	0.0149 (3)	
H4B	0.597271	0.664586	−0.073966	0.018*	
C5B	0.51371 (10)	0.67861 (11)	0.11794 (16)	0.0141 (3)	
H5B	0.478373	0.743923	0.082900	0.017*	
C6B	0.49478 (10)	0.62917 (11)	0.26090 (16)	0.0121 (3)	
H6B	0.445749	0.660464	0.323699	0.015*	
C7B	0.77056 (12)	0.33890 (13)	0.1449 (2)	0.0230 (3)	
H7BA	0.729477	0.327171	0.041043	0.035*	
H7BB	0.810613	0.272618	0.174984	0.035*	
H7BC	0.817418	0.400922	0.136102	0.035*	

### Atomic displacement parameters (Å^2^)

**Table:**

	*U*^11^	*U*^22^	*U*^33^	*U*^12^	*U*^13^	*U*^23^
S1A	0.01446 (17)	0.01894 (19)	0.01851 (19)	−0.00155 (13)	−0.00094 (13)	−0.00187 (13)
N1A	0.0110 (5)	0.0196 (6)	0.0125 (5)	0.0009 (4)	0.0008 (4)	−0.0003 (4)
C1A	0.0098 (6)	0.0203 (7)	0.0100 (6)	−0.0002 (5)	0.0022 (5)	0.0005 (5)
C2A	0.0102 (6)	0.0199 (7)	0.0111 (6)	−0.0010 (5)	0.0032 (5)	−0.0020 (5)
C3A	0.0129 (6)	0.0205 (7)	0.0155 (7)	0.0018 (5)	0.0011 (5)	0.0019 (5)
C4A	0.0112 (6)	0.0267 (7)	0.0139 (6)	0.0008 (5)	−0.0013 (5)	0.0017 (6)
C5A	0.0136 (6)	0.0250 (7)	0.0124 (6)	−0.0033 (5)	0.0013 (5)	−0.0021 (5)
C6A	0.0141 (6)	0.0194 (7)	0.0128 (6)	−0.0005 (5)	0.0029 (5)	0.0000 (5)
C7A	0.0238 (7)	0.0182 (7)	0.0215 (7)	−0.0003 (6)	0.0026 (6)	0.0015 (6)
S1B	0.01542 (17)	0.01406 (17)	0.01718 (18)	0.00275 (12)	0.00642 (13)	0.00248 (12)
N1B	0.0103 (5)	0.0140 (5)	0.0090 (5)	−0.0019 (4)	0.0012 (4)	−0.0012 (4)
C1B	0.0097 (5)	0.0135 (6)	0.0088 (6)	−0.0028 (5)	0.0008 (5)	−0.0014 (5)
C2B	0.0096 (5)	0.0112 (6)	0.0115 (6)	−0.0013 (5)	0.0001 (5)	−0.0013 (5)
C3B	0.0124 (6)	0.0176 (6)	0.0111 (6)	−0.0014 (5)	0.0042 (5)	−0.0018 (5)
C4B	0.0160 (6)	0.0180 (7)	0.0108 (6)	−0.0030 (5)	0.0020 (5)	0.0019 (5)
C5B	0.0142 (6)	0.0146 (6)	0.0130 (6)	0.0002 (5)	0.0000 (5)	0.0011 (5)
C6B	0.0109 (6)	0.0148 (6)	0.0106 (6)	−0.0011 (5)	0.0011 (5)	−0.0020 (5)
C7B	0.0229 (7)	0.0210 (7)	0.0276 (8)	0.0061 (6)	0.0144 (6)	0.0013 (6)

### Geometric parameters (Å, º)

**Table:**

S1A—C2A	1.7574 (14)	S1B—C2B	1.7605 (13)
S1A—C7A	1.8002 (15)	S1B—C7B	1.7983 (15)
N1A—N1A^i^	1.255 (2)	N1B—N1B^ii^	1.264 (2)
N1A—C1A	1.4211 (17)	N1B—C1B	1.4205 (16)
C1A—C2A	1.4080 (19)	C1B—C2B	1.4145 (18)
C1A—C6A	1.3940 (19)	C1B—C6B	1.3981 (18)
C2A—C3A	1.4046 (18)	C2B—C3B	1.3983 (18)
C3A—H3A	0.9500	C3B—H3B	0.9500
C3A—C4A	1.383 (2)	C3B—C4B	1.3866 (19)
C4A—H4A	0.9500	C4B—H4B	0.9500
C4A—C5A	1.393 (2)	C4B—C5B	1.3961 (19)
C5A—H5A	0.9500	C5B—H5B	0.9500
C5A—C6A	1.3857 (19)	C5B—C6B	1.3824 (19)
C6A—H6A	0.9500	C6B—H6B	0.9500
C7A—H7AA	0.9800	C7B—H7BA	0.9800
C7A—H7AB	0.9800	C7B—H7BB	0.9800
C7A—H7AC	0.9800	C7B—H7BC	0.9800
			
C2A—S1A—C7A	101.82 (7)	C2B—S1B—C7B	103.00 (7)
N1A^i^—N1A—C1A	113.98 (14)	N1B^ii^—N1B—C1B	114.53 (14)
C2A—C1A—N1A	115.37 (12)	C2B—C1B—N1B	115.83 (11)
C6A—C1A—N1A	123.50 (13)	C6B—C1B—N1B	124.14 (11)
C6A—C1A—C2A	121.11 (12)	C6B—C1B—C2B	120.02 (12)
C1A—C2A—S1A	118.27 (10)	C1B—C2B—S1B	118.09 (10)
C3A—C2A—S1A	123.84 (11)	C3B—C2B—S1B	123.24 (10)
C3A—C2A—C1A	117.89 (12)	C3B—C2B—C1B	118.67 (12)
C2A—C3A—H3A	119.8	C2B—C3B—H3B	119.8
C4A—C3A—C2A	120.33 (13)	C4B—C3B—C2B	120.46 (12)
C4A—C3A—H3A	119.8	C4B—C3B—H3B	119.8
C3A—C4A—H4A	119.3	C3B—C4B—H4B	119.6
C3A—C4A—C5A	121.35 (13)	C3B—C4B—C5B	120.79 (12)
C5A—C4A—H4A	119.3	C5B—C4B—H4B	119.6
C4A—C5A—H5A	120.4	C4B—C5B—H5B	120.3
C6A—C5A—C4A	119.12 (13)	C6B—C5B—C4B	119.42 (13)
C6A—C5A—H5A	120.4	C6B—C5B—H5B	120.3
C1A—C6A—H6A	119.9	C1B—C6B—H6B	119.7
C5A—C6A—C1A	120.10 (13)	C5B—C6B—C1B	120.63 (12)
C5A—C6A—H6A	119.9	C5B—C6B—H6B	119.7
S1A—C7A—H7AA	109.5	S1B—C7B—H7BA	109.5
S1A—C7A—H7AB	109.5	S1B—C7B—H7BB	109.5
S1A—C7A—H7AC	109.5	S1B—C7B—H7BC	109.5
H7AA—C7A—H7AB	109.5	H7BA—C7B—H7BB	109.5
H7AA—C7A—H7AC	109.5	H7BA—C7B—H7BC	109.5
H7AB—C7A—H7AC	109.5	H7BB—C7B—H7BC	109.5
			
S1A—C2A—C3A—C4A	−177.02 (11)	S1B—C2B—C3B—C4B	−179.51 (10)
N1A^i^—N1A—C1A—C2A	−168.19 (14)	N1B^ii^—N1B—C1B—C2B	175.48 (13)
N1A^i^—N1A—C1A—C6A	13.2 (2)	N1B^ii^—N1B—C1B—C6B	−5.3 (2)
N1A—C1A—C2A—S1A	−3.06 (16)	N1B—C1B—C2B—S1B	−2.06 (15)
N1A—C1A—C2A—C3A	177.45 (12)	N1B—C1B—C2B—C3B	178.30 (11)
N1A—C1A—C6A—C5A	−178.94 (12)	N1B—C1B—C6B—C5B	−178.56 (12)
C1A—C2A—C3A—C4A	2.4 (2)	C1B—C2B—C3B—C4B	0.11 (19)
C2A—C1A—C6A—C5A	2.5 (2)	C2B—C1B—C6B—C5B	0.61 (19)
C2A—C3A—C4A—C5A	0.3 (2)	C2B—C3B—C4B—C5B	1.1 (2)
C3A—C4A—C5A—C6A	−1.8 (2)	C3B—C4B—C5B—C6B	−1.4 (2)
C4A—C5A—C6A—C1A	0.4 (2)	C4B—C5B—C6B—C1B	0.54 (19)
C6A—C1A—C2A—S1A	175.63 (10)	C6B—C1B—C2B—S1B	178.71 (10)
C6A—C1A—C2A—C3A	−3.86 (19)	C6B—C1B—C2B—C3B	−0.94 (18)
C7A—S1A—C2A—C1A	−176.89 (11)	C7B—S1B—C2B—C1B	−179.91 (10)
C7A—S1A—C2A—C3A	2.57 (13)	C7B—S1B—C2B—C3B	−0.28 (13)

Symmetry codes: (i) −*x*, −*y*+1, −*z*; (ii) −*x*+1, −*y*+1, −*z*+1.
